# Supplementary material for: State-behaviour feedbacks between boldness and food intake shape escape responses in fish (Gasterosteus aculeatus)
Source: Commun Biol. 2025 Feb 13;8:227. doi: 10.1038/s42003-025-07669-w (PMC11825722; doi:10.1038/s42003-025-07669-w)
Supplement: Supplementary file 2 — Supplementary information [file 42003_2025_7669_MOESM2_ESM.pdf]

State-behaviour feedbacks between boldness and food intake shape escape responses in fish (*Gasterosteus aculeatus*)

Isaac Planas-Sitjà[1]* & Christos. C. Ioannou[2]

[1] Department of Biological Sciences, Tokyo Metropolitan University, 1-1 Minami-Osawa, Hachioji, Tokyo, 192-0397, Japan; [2] School of Biological Sciences, University of Bristol, Life Sciences Building, 24 Tyndall Avenue, Bristol BS8 1TQ, United Kingdom; *corresponding author: iplanass@pm.me

Journal: Communications Biology

Supplementary material

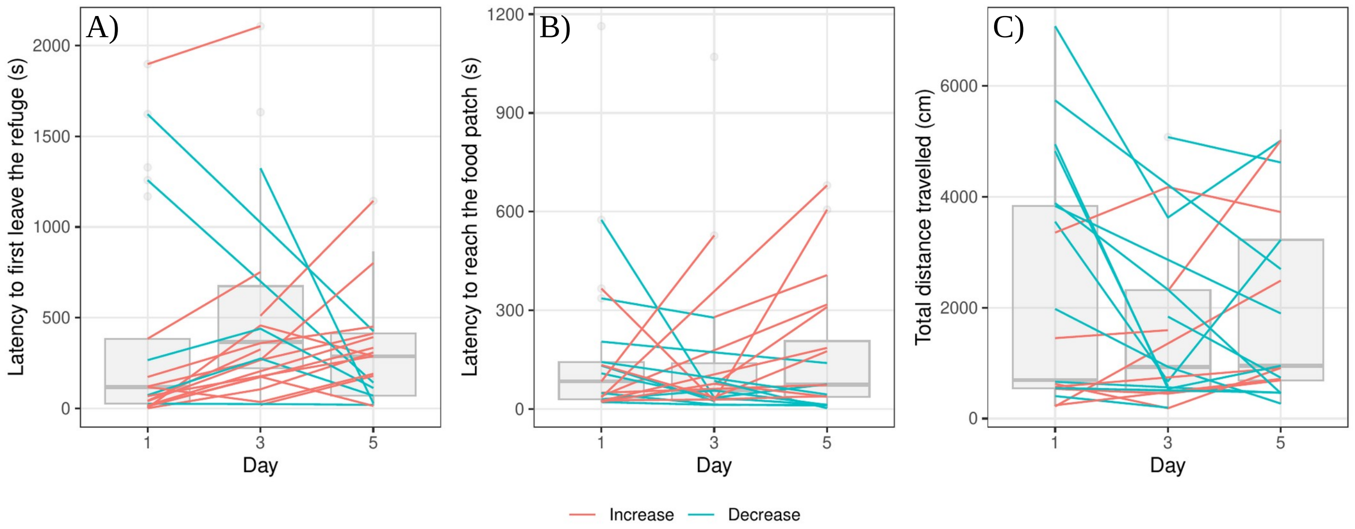

Supplementary figure 1: Reaction norms. Change in latency to first leave the refuge (A), latency to reach the food patch (B) and total distance travelled (C) over three trial days. Boxplots indicate the distribution for each day, while lines indicate individual changes.

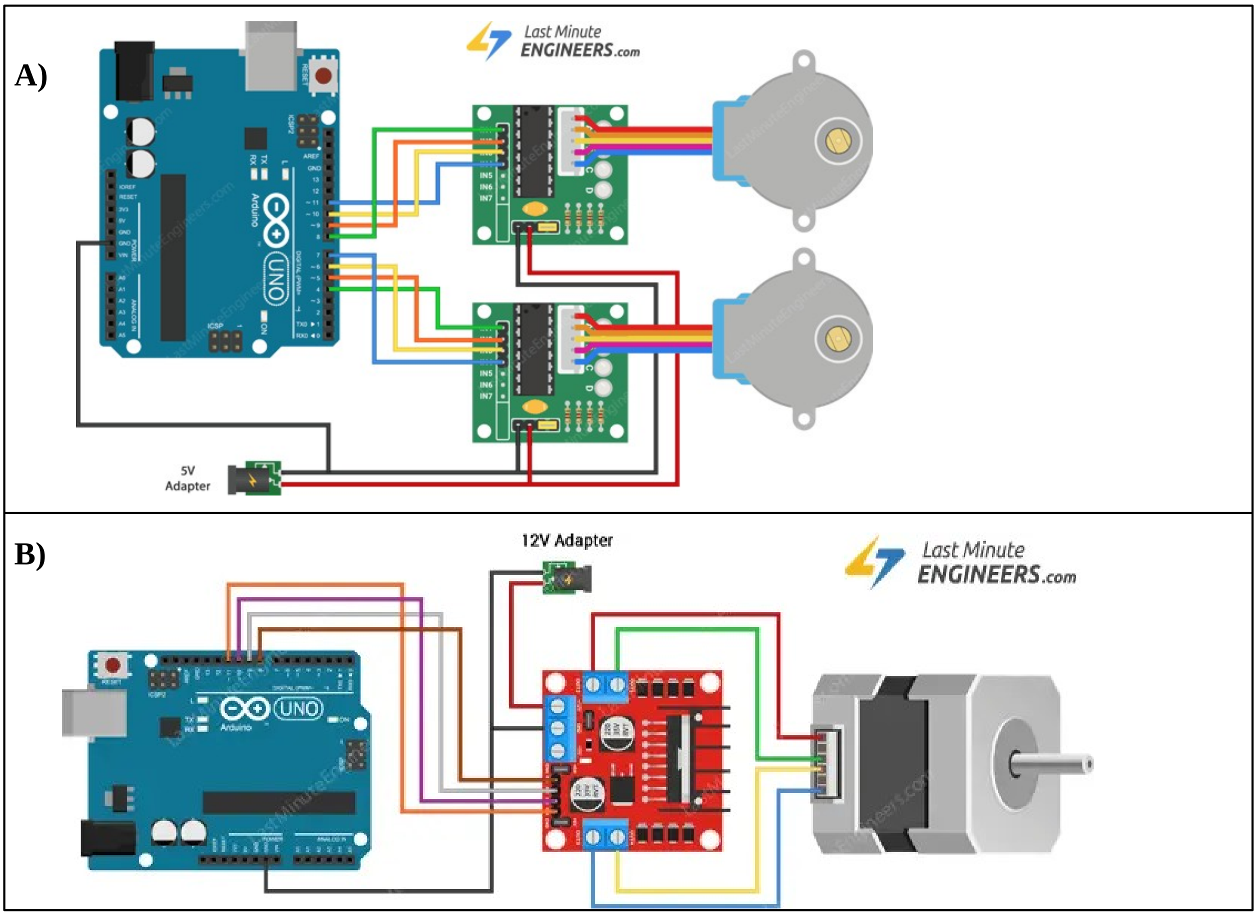

Supplementary figure 2: Circuit diagrams. A) Circuit diagram used to open/close the refuge doors, two doors at the same time (image from https://lastminuteengineers.com/28byj48-stepper-motor-arduino-tutorial/). B) Circuit diagram used to trigger the heron model (image from https://lastminuteengineers.com/stepper-motor-l298n-arduino-tutorial/)

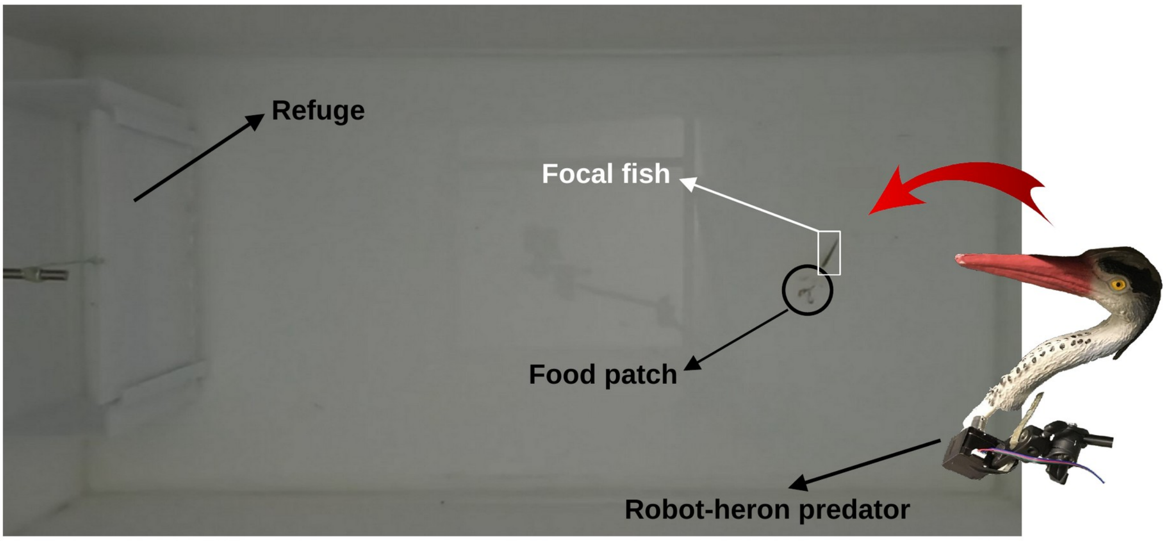

Supplementary figure 3. Diagram of the setup. The robot-heron predator was activated when the focal fish entered inside the food patch.

Supplementary table 1: GLMM1. Most parsimonious model highlighted in bold and with asterisks. Table shows the five models with the lowest AICc (or all models with a dAICc lower than 2.0 if those are not included within the five models), and the model with the highest dAICc (bottom).

**Response variable: Freeze or flee (Binomial)**	dAICc	df
BW before + Distance predator	0.0	4
BW before	0.2	3
BW before + Angle attack + Distance predator	0.2	5
Null model	1.3	2
Angle attack + Distance predator	1.6	4
BW before + Angle attack	1.8	4
BW before + Body length	1.9	4
Distance predator	2.0	3
BW before +Distance predator + Body length	2.0	5
...		
Latency * BW before + Body length + Day + Angle attack	9.3	8

Full model: ~ Latency + BW before + Latency:BW before + Body length + Day + Angle attack + Distance predator + (1|Individual ID) [dAICc = 9.2]
Null model: ~ (1|Individual ID) [dAICc = 1.3]

<u>Description of fixed effects</u>

- Latency = latency to first leave refuge (boldness)
- BW before = number of bloodworms eaten before attack (food intake)
- Latency:BW before = interaction between boldness and food intake
- Body length = body length of the fish
- Day = trial day (1, 2, 3)
- Angle attack = angle (direction) from the attack
- Distance predator = distance from the predator

Supplementary table 2: Summary of the most parsimonious model for GLMM1.

	Estimate	SD	z value
(Intercept)	0.849	0.527	1.074
BW before	-0.67	0.397	-1.686

Test of residuals (DHARMa package)

	p-value
KS test p-value	0.95
Dispersion test	0.77
Outlier test	1

Supplementary table 3: GLMM2. Most parsimonious model highlighted in bold and with asterisks. Table shows the five models with the lowest AICc (or all models with a dAICc lower than 2.0 if those are not included within the five models), and the model with the highest dAICc (bottom).

**Response variable: Escape latency (Gaussian)**	dAICc	df
Latency + BW before + Day + Angle attack	0.0	7
Latency + BW before	1.8	5
Latency + BW before + Day	1.9	6
Latency + BW before + Body length + Day + Distance predator	2.1	8
Latency + BW before + Body length + Day + Angle attack	2.1	8
…		
BW before + Body length + Distance predator + Angle attack	12.9	7

Full model: ~ Latency + BW before + Latency:BW before + Body length + Day + Angle attack + Distance predator + (1|Individual ID) [dAICc = 7.3]
Null model: ~ (1|Individual ID) [dAICc = 9.3]

Description of fixed effects

- Latency = latency to first leave refuge (boldness)
- BW before = number of bloodworms eaten before attack (food intake)
- Latency:BW before = interaction between boldness and food intake
- Body length = body length of the fish
- Day = trial day (1, 2, 3)
- Angle attack = angle (direction) from the attack
- Distance predator = distance from the predator

Supplementary table 4: Summary of the most parsimonious model for GLMM2.

	Estimate	SD	t value
(Intercept)	0.693	0.116	5.995
Latency	0.177	0.065	2.711
BW before	0.164	0.057	2.906
Day	0.098	0.033	3.006
Angle attack	0.149	0.065	2.300

Test of residuals (DHARMa package)

	p-value
KS test	0.15
Dispersion test	0.84
Outlier test	1

Supplementary table 5: GLMM3. Most parsimonious model highlighted in bold and with asterisks. Table shows the five models with the lowest AICc (or all models with a dAICc lower than 2.0 if those are not included within the five models), and the model with the highest dAICc (bottom).

**Response variable: Turn rate (Gaussian)**	dAICc	df
Distance predator + Curvature	0.0	5
Body length + Distance predator + Curvature	0.5	6
Latency + Distance predator + Curvature	0.7	7
Curvature	1.3	4
Body length + Curvature	1.4	5
BW before + Body length + Distance predator + Curvature	1.6	6
BW before + Angle attack + Distance predator + Curvature	1.9	8
BW before + Body length + Curvature	2.2	6
…		
Latency * BW before + Body length + Day + Angle attack + Distance predator	30.6	10

Full model: ~ Latency + BW before + Latency:BW before + Body length + Day + Angle attack + Distance predator + Curvature + (1|Individual ID) [dAICc = 10.9]
Null model: ~ (1|Individual ID) [dAICc = 13.8]

Description of fixed effects

- Latency = latency to first leave refuge (boldness)
- BW before = number of bloodworms eaten before attack (food intake)
- Latency:BW before = interaction between boldness and food intake
- Body length = body length of the fish
- Day = trial day (1, 2, 3)
- Angle attack = angle (direction) from the attack
- Distance predator = distance from the predator
- Turn rate = turn rate during escape
- Curvature = curvature index, maximum bend of the body during escape (lower index = higher curvature)

Supplementary table 6: Summary of the most parsimonious model for GLMM3

	Estimate	SD	t value
(Intercept)	2664.71	104.66	25.46
Curvature	-400.55	82.16	-4.875
Distance predator	146.76	71.87	2.042

Test of residuals (DHARMa package)

	p-value
KS test	0.72
Dispersion test	0.65
Outlier test	1

Supplementary table 7: GLMM4. Most parsimonious model highlighted in bold and with asterisks. Table shows the five models with the lowest AICc (or all models with a dAICc lower than 2.0 if those are not included within the five models), and the model with the highest dAICc (bottom).

**Response variable: Curvature index (Gaussian)**	dAICc	df
Distance predator + Turn rate	0.0	5
Latency + Distance predator + Turn rate	1.9	6
Angle attack + Distance predator + Turn rate	2.2	6
Day + Distance predator + Turn rate	2.6	6
BW before + Distance predator + Turn rate	2.7	6
…		
Latency * BW before + Body length + Day + Angle attack + Distance predator	33.8	10

Full model: ~ Latency + BW before + Latency:BW before + Body length + Day + Angle attack + Distance predator + Turn rate + (1|Individual ID) [dAICc = 16.9]
Null model: ~ (1|Individual ID) [dAICc = 17.7]
Description of fixed effects

- Latency = latency to first leave refuge (boldness)
- BW before = number of bloodworms eaten before attack (food intake)
- Latency:BW before = interaction between boldness and food intake
- Body length = body length of the fish
- Day = trial day (1, 2, 3)
- Angle attack = angle (direction) from the attack
- Distance predator = distance from the predator
- Turn rate = turn rate during escape
- Curvature = curvature index, maximum bend of the body during escape (lower index = higher curvature)

Supplementary table 8: Summary of the most parsimonious model for GLMM4

	Estimate	SD	t value
(Intercept)	0.647	0.010	62.042
Turn rate	-0.053	0.010	-5.183
Distance predator	0.022	0.009	2.475

Test of residuals (DHARMa package)

	p-value
KS test	0.95
Dispersion test	0.8
Outlier test	1

Supplementary table 9: GLMM5. Most parsimonious model highlighted in bold and with asterisks. Table shows the five models with the lowest AICc (or all models with a dAICc lower than 2.0 if those are not included within the five models), and the model with the highest dAICc (bottom).

**Response variable: Initial speed (Gaussian)**	dAICc	df
Latency + Body length	0	5
Latency + Body length + Day	0.2	6
Null model	0.6	4
Curvature	0.7	5
Latency + Body length + Curvature	0.8	6
BW before + Body length + Turn rate	1.4	6
BW before + Body length + Angle attack	1.6	6
BW before + Body length + Day + Curvature	1.6	7
Body length + Turn rate	1.8	5
Body length + Day	1.9	5
Latency + Body length	2	5
…		
Latency * BW before + Day + Angle attack + Distance predator + Turn rate + Curvature	19.2	11

Full model: ~ Latency + BW before + Latency:BW before + Body length + Day + Angle attack + Distance predator + Turn rate + Curvature + (1|Individual ID) [dAICc = 18.9]
Null model: ~ (1|Individual ID) [dAICc = 0.6]

Description of fixed effects

- Latency = latency to first leave refuge (boldness)
- BW before = number of bloodworms eaten before attack (food intake)
- Latency:BW before = interaction between boldness and food intake
- Body length = body length of the fish
- Day = trial day (1, 2, 3)
- Angle attack = angle (direction) from the attack
- Distance predator = distance from the predator
- Turn rate = turn rate during escape
- Curvature = curvature index, maximum bend of the body during escape (lower index = higher curvature)

Supplementary table 10: GLMM6. Most parsimonious model highlighted in bold and with asterisks. Table shows the five models with the lowest AICc (or all models with an dAICc lower than 2.0 if those are not included within the five models), and the model with the highest dAICc (bottom).

**Response variable: Recovery time (Poisson)**	dAICc	df
Latency + BW before + Body length + Day + Escape distance	**0.0**	**7**
Latency + BW before + Body length + Day + Escape distance + Distance refuge	1.3	8
Latency * BW before + Body length + Day + Escape distance	2.2	8
Latency * BW before + Body length + Day + Escape distance + Distance refuge	3.5	9
Latency + BW before + Body length + Escape distance + Distance refuge	20.6	7
…		
BW before	1130.5	3

Full model: ~ Latency + BW before + Latency:BW before + Body length + Day + Escape distance + Distance refuge + (1|Individual ID) [dAICc = 3.5]
Null model: ~ (1|Individual ID) [dAICc = 1128.4]

<u>Description of fixed effects</u>

- Latency = latency to first leave refuge (boldness)
- BW before = number of bloodworms eaten before attack (food intake)
- Latency:BW before = interaction between boldness and food intake
- Body length = body length of the fish
- Day = trial day (1, 2, 3)
- Escape distance = distance travelled during escape, from the initial movement to full stop (assessed by visual inspection)
- Distance refuge = distance from the refuge when fish stop after escaping (if they flee) or at the position they froze (if freeze).

Supplementary table 11: Summary of the most parsimonious model for GLMM6

	Estimate	SD	z value
(Intercept)	4.853	0.164	29.540
Latency	0.161	0.020	7.778
BW before	0.196	0.018	10.875
Day	0.033	0.007	4.942
Escape distance	0.589	0.019	30.255
Body length	-0.422	0.067	-6.279

Test of residuals (DHARMa package)

	p-value
KS test	0.36
Dispersion test	0.39
Outlier test	0.24
